# Structural dissection of vaccinia G9 identifies residues essential for membrane fusion and complex assembly

**DOI:** 10.1128/jvi.00723-25

**Published:** 2025-08-18

**Authors:** Hsiao-Jung Chiu, Hao-Ching Wang, Wen Chang

**Affiliations:** 1 Molecular and Cell Biology, Taiwan International Graduate Program, Academia Sinica and Graduate Institute of Life Sciences, National Defense Medical Center 71548, Taipei, Taiwan; 2 Institute of Molecular Biology 71562, Taipei, Taiwan; 3 The PhD Program for Translational Medicine, College of Medical Science and Technology, Taipei Medical University and Academia Sinica 38032, Taipei, Taiwan; 4 Graduate Institute of Translational Medicine, College of Medical Science and Technology, Taipei Medical University 38032, Taipei, Taiwan; Northwestern University Feinberg School of Medicine, Chicago, Illinois, USA

**Keywords:** vaccinia virus, EFC, G9 protein, membrane fusion, poxvirus entry

## Abstract

Vaccinia virus, a prototypical poxvirus, utilizes a unique multi-protein entry fusion complex (EFC), comprising 11 components, to mediate membrane fusion during host cell entry. Although the crystal structure of a truncated form of the G9 protein has been determined, the functional relevance of its structural features remains poorly understood. In this study, we systematically analyzed 47 G9 mutants to identify critical functional residues. Using trans-complementation assays, co-immunoprecipitation, membrane fusion assays, and structural analysis, we identified nine key mutants, which were categorized into three functional groups. Group 1 mutants failed to interact with A16 and other EFC components, highlighting their essential roles in G9-A16 subcomplex formation. Group 2 and Group 3 mutants retained A16 binding but disrupted interactions with other EFC proteins, suggesting their roles in broader complex assembly. Notably, Group 3 mutants targeted a conserved P(R/Y)XCW motif and a loop structure shared among vaccinia G9, A16, and J5 proteins. A similar motif was also identified in G9 homologs from *Nucleocytoviricota*, suggesting an evolutionarily conserved fusion mechanism. Collectively, our findings demonstrated that G9 function requires multiple domains, including A16-binding interfaces and conserved motifs not resolved in previous protein structures. These results establish G9 as a central EFC component and underscore its potential as a target for antiviral development.

**IMPORTANCE:**

Understanding how viruses enter host cells is critical for developing antiviral strategies. Vaccinia virus, a model poxvirus, uses a unique 11-protein entry fusion complex (EFC) to mediate membrane fusion, unlike other viruses that rely on a single fusion protein. In this study, we identified specific residues in the G9 protein that are critical for maintaining EFC function. Notably, we discovered a conserved P(R/Y)XCW motif within G9 that is also present in orthologs from both poxviruses and members of the *Nucleocytoviricota* phylum, suggesting an evolutionarily conserved mechanism of membrane fusion. These conserved structural elements can serve as potential targets for antiviral intervention against pathogenic poxvirus infections in humans.

## INTRODUCTION

Vaccinia virus is a large double-stranded DNA virus belonging to the genus *Orthopoxvirus* in the *Poxviridae* family (1). Its genome is approximately 190 kbp in length and encodes over 200 proteins (2). Vaccinia virus contains two infectious forms of virus particles produced from the infected cells, the mature virus (MV) and the enveloped virus (EV). The majority of the virus particles (>95%) accumulated intracellularly as MV, which are brick-shaped with membranes derived from the endoplasmic reticulum (3, 4). A small fraction of MV (~5%) is transported to the Golgi apparatus, where they acquire two additional membrane layers derived from the Golgi cisternae, forming wrapped virions (WVs) (5, 6). These WVs are transported along microtubules to the cell periphery (7, 8), where they fuse in an inside-out manner with the plasma membrane, releasing extracellular EV (9, 10).

Vaccinia MV contains four attachment proteins: H3 (11), D8 (12), A27 (13), and A26 (14). Membrane fusion is mediated by an 11-protein entry fusion complex (EFC), composed of G9 (15), A16 (16), J5 (17), A21 (18), A28 (19), H2 (20), G3 (21), L5 (22), F9 (23), L1 (24), and O3 (25), all of which are highly conserved across the *Poxviridae* family (26). Deletion or inhibition of any individual EFC component resulted in defective membrane fusion, demonstrating that each is essential for EFC function (26). Although the EFC becomes destabilized when specific components are lost, subunit interactions such as G9-A16, H2-A28, and G3-L5 remain stable (27–29). Recent studies suggested that L1 and F9 interact weakly with the rest of the complex, indicating they may serve as the peripheral rather than core components (23, 24, 30). Notably, while individual EFC proteins contribute to distinct stages of the membrane fusion process, G9, A16, A21, H2, G3, F9, and O3 appear to function in the initiation of hemifusion, whereas A28, L1, and L5 act in post-hemifusion steps (31). Although the ectodomain structures of nearly all EFC components, except J5 and O3, have been resolved (32–38), the molecular mechanism of EFC-mediated membrane fusion remains poorly understood.

To regulate membrane fusion, vaccinia virus encodes fusion suppressors that keep the activity of the EFC under control. One such suppressor, the A26 protein, directly binds to the G9-A16 subcomplex and inhibits premature fusion activation at neutral pH (39). Another suppressor complex, composed of A56 and K2, is expressed on the surface of infected cells and also interacts with the G9-A16 subcomplex to prevent superinfection (30). Interestingly, experimental evolutionary studies have shown that a single H44Y mutation in the G9 protein can overcome A56/K2-mediated inhibition, enabling membrane fusion to occur at pH 6 instead of pH 5 (30). Furthermore, phylogenetic analyses revealed that G9 and A16 homologs of the EFC are present in *Nucleocytoviricota*, implying that the vaccinia EFC evolved from a shared ancestral fusion machinery (40). Given the essential role of G9 protein in EFC-mediated membrane fusion, we sought to dissect its structural and functional relationship. Using site-directed mutagenesis, we identified critical regions of G9 required for viral infectivity, subcomplex formation, and assembly of an intact EFC. These findings provide structural insights into the contribution of G9 protein to membrane fusion in vaccinia virus.

## MATERIALS AND METHODS

### Cell culture and virus

Human 293T and African green monkey kidney BSC40 cells were maintained in Dulbecco’s modified Eagle medium supplemented with 10% fetal bovine serum (36). Recombinant vaccinia virus vG9Li-HA, which expresses the vaccinia G9 protein under induction with isopropyl-β-d-thiogalactopyranoside (IPTG), was kindly provided by Bernard Moss (15). The vG9Li-HA virus was propagated in BSC40 cells cultured in medium supplemented with 50 µM IPTG, as previously described (15).

### Construction of G9 expression plasmids by site-directed mutagenesis

The vaccinia G9R gene, tagged at the N-terminus with a c-myc epitope, was cloned into mammalian expression vectors pCAGEN or pcDNA3.1 to generate pCAGEN-myc-G9R or pcDNA3.1-myc-G9R. Mutant G9 constructs were generated using the QuikChange Lightning Site-Directed Mutagenesis Kit (Agilent) based on either of the two plasmids. All constructs were sequence-verified (Genomics Inc., Taiwan).

### Trans-complementation assays

Trans-complementation assays were performed as previously described (36). Briefly, 293T cells were seeded in six-well plates and transfected with 1 µg of wild-type (WT) or mutant G9 plasmid using 10 µL Lipofectamine 2000 (Invitrogen). After 24 h, the cells were infected with vG9Li-HA at a multiplicity of infection (MOI) of 1 PFU/cell at 37°C for 1 h, followed by incubation in growth medium without IPTG. At 24 h post-infection (hpi), the cells were harvested for immunoblot and plaque assays. Each mutant was tested in three independent experiments. The mutant experiments were conducted as several smaller, independent experiments. In each case, the same controls were consistently included: vector-transfected/vG9Li-HA-infected cells with or without IPTG, and WT G9 plasmid-transfected/vG9Li-HA-infected cells.

### Co-immunoprecipitation of G9 and EFC components

BSC40 cells in 100 mm dishes were infected with vG9Li-HA at an MOI of 5 PFU/cell for 1 h at 37°C, followed by transfection with 0.5 µg of WT or mutant G9 plasmid using 20 µL Lipofectamine 2000 in IPTG-free medium. At 24 hpi, the cells were lysed on ice in 500 µL of lysis buffer (0.5% NP-40, 20 mM Tris-HCl, pH 8.0, and 200 mM NaCl) supplemented with protease inhibitors (2 µg/mL aprotinin, 1 µg/mL leupeptin, 0.7 µg/mL pepstatin, and 1 mM PMSF). Lysates were clarified by centrifugation at 16,000 × *g* for 15 min. Supernatants were incubated with c-myc-conjugated agarose beads (Sigma-Aldrich) at 4°C overnight and analyzed by immunoblotting with anti-EFC component antibodies (36). Each experiment was independently performed two times.

### MV-triggered cell-cell fusion at acidic pH

Cell-cell fusion assays were conducted as described previously (36). HeLa-RFP and HeLa-GFP cells were co-seeded in 96-well plates at a 1:1 ratio and treated with 40 µg/mL cordycepin for 1 h at 37°C before infection. WT or mutant G9 virus-containing lysates (from trans-complementation assays) were incubated with cells for 1 h at 37°C. The cells were then washed and exposed to pH 7.0 or 5.0 buffer at 37°C for 3 min, neutralized with growth medium, and incubated with cordycepin-containing medium for 3 h. Images were acquired from 30 min to 3 hpi using an ImageXpress Confocal HT.ai High Content Imaging System. Fusion efficiency was quantified using Fiji software as: (surface area of GFP^+^RFP^+^ double-fluorescent cells/surface area of single-fluorescent cells) × 100%, as previously described (35). WT G9-triggered fusion at low pH was set at 100%, and values for each mutant were normalized accordingly. All the experiments were repeated three times.

### Electron microscopy (EM)

BSC40 cells were infected with vG9Li-HA at an MOI of 5 PFU per cell and transfected with 1 µg of WT or mutant G9 plasmid using 10 µL Lipofectamine 2000 (Invitrogen). At 24 hpi, the cells were fixed with glutaraldehyde and followed by secondary fixation with osmium tetroxide. The samples were stained with uranyl acetate, dehydrated, and embedded in Epon resin for sectioning as described (39). Sample sections were imaged using a Tecnai G2 Spirit TWIN transmission electron microscope.

### Multiple sequence alignments of G9 orthologues in *Poxviridae* and *Nucleocytoviricota*

Orthologs of vaccinia G9 protein from various *Poxviridae* members (e.g., VACV, CMLV, CPXV, ECTV, and MPXV) were retrieved from GenBank. Accession numbers and virus abbreviations are listed: vaccinia virus (YP_232969.1); CMLV: camelpox virus (NP_570475.1); CPXV: cowpox virus (NP_619884.1); ECTV: ectromelia virus (NP_671589.1); MPXV: monkeypox virus (NP_536506.1); SKPV: skunkpox virus (YP_009282780.1); TATV: taterapox virus (YP_717397.1); VARV: variola virus (NP_042116.1); VPXV: volepox virus (YP_009281834.1); EWPV: fowlpox virus (NP_039090.1); TKPV: turkeypox virus (YP_009177111.1); GTPV: goatpox virus (YP_001293250.1); YKPV: yokapox virus (YP_004821420.1); CRPV: crocodilepox virus (QGT49326.1); SQPV: squirrelpox virus (YP_008658478.1); MYXV: myxoma virus (NP_051768.1); MCPV: molluscum contagiosum virus (NP_044019.1); SEPV: sea otter poxvirus (YP_009480597.1); BPSV: bovine papular stomatitis virus (NP_957955.1); RDPV: red deer parapoxvirus (YP_009112785.1); PCPV: pseudocowpox virus (YP_003457351.1); ORFV: orf virus (QLI57553); PTPV: pteropox virus (YP_009268779.1); SGPV: salmon gill poxvirus (AKR04265.1); YMTV: yaba monkey tumor virus (NP_938314.1); ACEV: anomala cuprea entomopoxvirus (YP_009001529.1); EPTV: eptesipox virus (YP_009408014.1); and AMEV: amsacta moorei entomopoxvirus (NP_064817.1). Multiple sequence alignments were generated using MAFFT v7.453 with parameters --maxiterate 1000 --globalpair, and visualized with Jalview v1.8.3. Residue conservation was color-coded: yellow (identical), blue (>0.5 conservation), and green (>0.2 conservation). Alignment of 11 G9 homologs genes from six *Nucleocytoviricota* members, Klosneuvirus (ARF12395.1, ARF12396.1), Frog virus 3 (YP_031580.1), Tupanvirus deep ocean (QKU34524.1), Medusavirus (BBI30243.1, BBI30251.1, BBI30253.1, BBI30262.1), Pacmanvirus A23 (YP_009361353.1, YP_009361420.1), and Invertebrate iridescent virus 6(NP_149800.1), also reveals a conserved motif. Sequences were aligned using MacVector (v16.0.10), and consensus logos were generated via WebLogo3 (http://weblogo.threeplusone.com/).

### Protein structure prediction with AlphaFold2

Full-length G9 (YP_232969.1), A16 (YP_233018.1), and J5 (YP_232979.1) structures were predicted using AlphaFold2 (https://reurl.cc/96anZj). Sequences were queried using MMseqs2 (mmseqs2_uniref_env) in unpaired/paired mode. The predicted model with a pLDDT score >70 was selected for each protein. Final confidence scores for G9, A16, J5, and the G9-A16 subcomplex were 86.9, 81.2, 80.7, and 78.3, respectively.

To assess the accuracy of AlphaFold2 multimer predictions, we examined the predicted template modeling (pTM) score. According to AlphaFold2 guidelines (https://reurl.cc/M3Q5ap), a pTM score above 0.5 indicates high confidence in the overall structural fold. The G9-A16 subcomplex yielded a pTM score of 0.752.

### Residue interaction analysis

The G9-A16 subcomplex structure (PDB: 8GP6) (38) was analyzed using the Residue Interaction Network Generator (RING; https://ring.biocomputingup.it/) to identify intra- and inter-subunit residue contacts.

### Statistical analysis

Statistical analyses were performed using Student’s *t*-test in GraphPad Prism v.9.2, as previously described (35). Data are presented as mean ± standard deviation (SD). For comparison with WT, the *P* value was adjusted using the “p.adjust” function with method “fdr” in R v4.5.1. The adjusted *P* value less than 0.05 was considered as statistically significant. *P < 0.05, **P < 0.01, and ***P < 0.001, and **** *P* < 0.0001.

## RESULTS

### Identifying the functional region of VACV G9 protein

**Fig 1:**
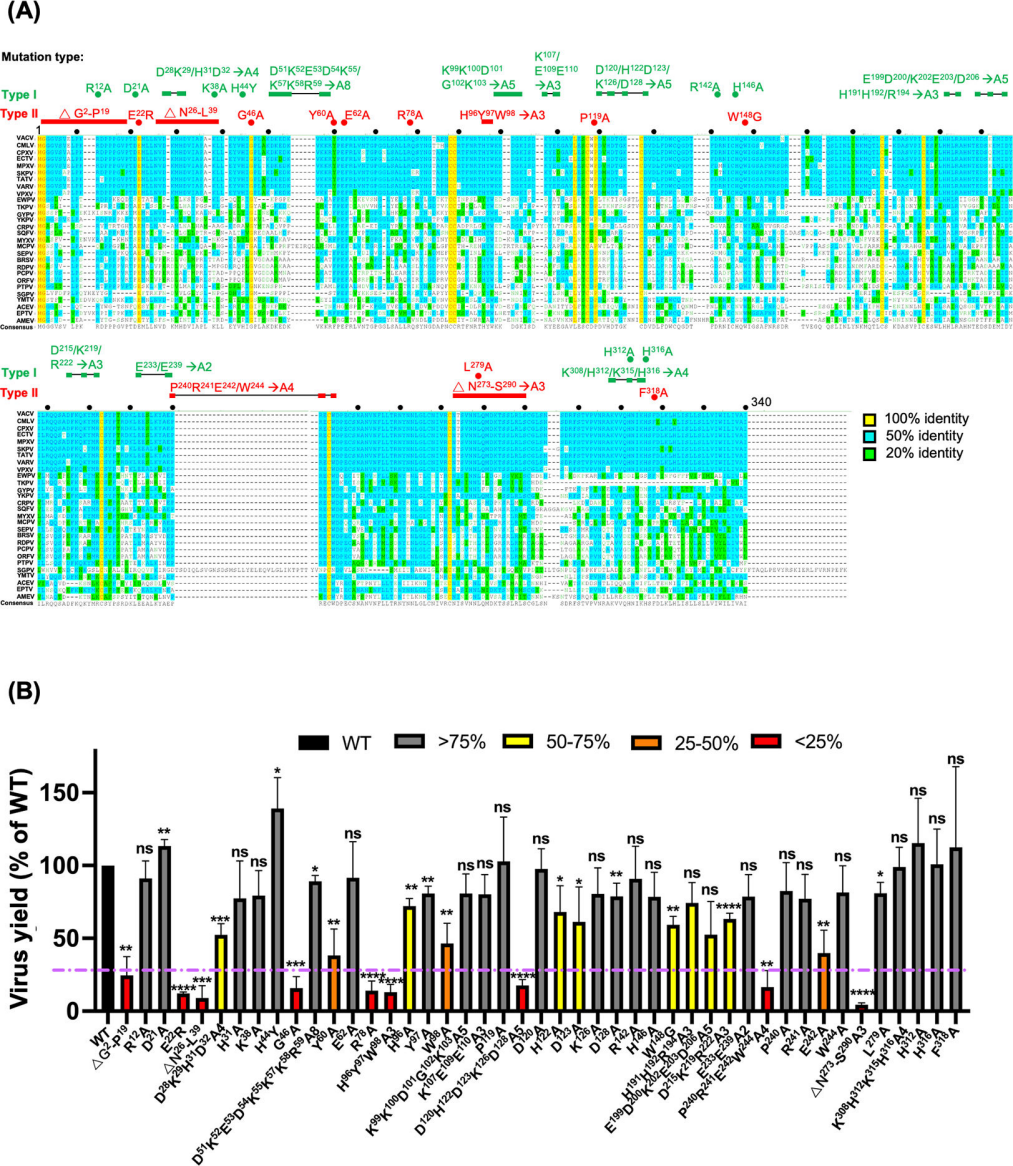
Identification of G9 critical regions important for vaccinia MV infectivity. (A) Two types of G9 mutations were depicted on top of the Multiple sequence alignment (MSA) of 28 G9 orthologues in *Poxviridae*: Mutations of the charged surface residues (type I) and conserved residues (type II) are shown in green and red, respectively. Colored dots and bridged boxes represent mutation sites. Black dots mark the distance of 10 a.a. in the MSA. (B) Virus yields of G9 mutants from trans-complementation assays. 293T cells were transfected with wild-type (WT) or mutant G9 plasmid and infected with vG9Li-HA and cultured for 24 h without IPTG and harvested at 24 hpi for plaque assays. The complementary assays were conducted as several smaller sets of experiments, each repeated three times independently. Each experiment included the same control, vector-transfected/vG9Li-HA-infected cells with or without IPTG, and WT G9 plasmid-transfected/vG9Li-HA-infected cells. The virus yield of complementation with WT-G9 plasmid was comparable in all experiments (~10-fold). The percentage of virus yield in each G9 mutant was normalized with that of WT G9. The purple dashed line indicates the 25% infectivity threshold. Student’s *t*-test: *P < 0.05, **P < 0.01, ***P < 0.001, and ****P < 0.0001.

To define the functional role of the vaccinia G9 protein during infection, we performed systematic mutagenesis of the G9 open reading frame (ORF), targeting two categories of residues: (i) surface-exposed charged residues (type I) and (ii) conserved residues among G9 orthologs in the *Poxviridae* (type II), as shown in Fig. 1A. A total of 47 mutant constructs were generated and evaluated for their ability to support virus replication using a trans-complementation assay in 293T cells. Plasmids expressing either WT or mutant G9 proteins were transfected into 293T cells 24 h prior to infection with the IPTG-inducible virus vG9Li-HA (15) at an MOI of 1 PFU/cell in the absence of IPTG. This setup ensures that G9 protein was only expressed from the transfected plasmid. At 24 hpi, the cells were harvested for plaque assays and immunoblot analyses. All 47 mutant proteins were successfully expressed (see immunoblots in Fig. 3A and B, 4A, 5A and B, and 6D and E). WT G9 plasmid complementation increased virus yield ~10-fold at 24 hpi, while IPTG induction produced yields two times as high. The complementary effect of the WT-G9 plasmid was set as 100% infectivity (Fig. 1B, black bar). In contrast, mutant constructs exhibited a wide range of phenotypes: eight mutants retained 50–75% infectivity (Fig. 1B, yellow bars), three showed 25–50% infectivity (Fig. 1B, orange bars), and nine had infectivity below 25% (Fig. 1B, red bars). The last group of nine low-infectivity mutants was selected for further functional characterization.

### G9 mutants with low MV infectivity are defective in membrane fusion

**Fig 2:**
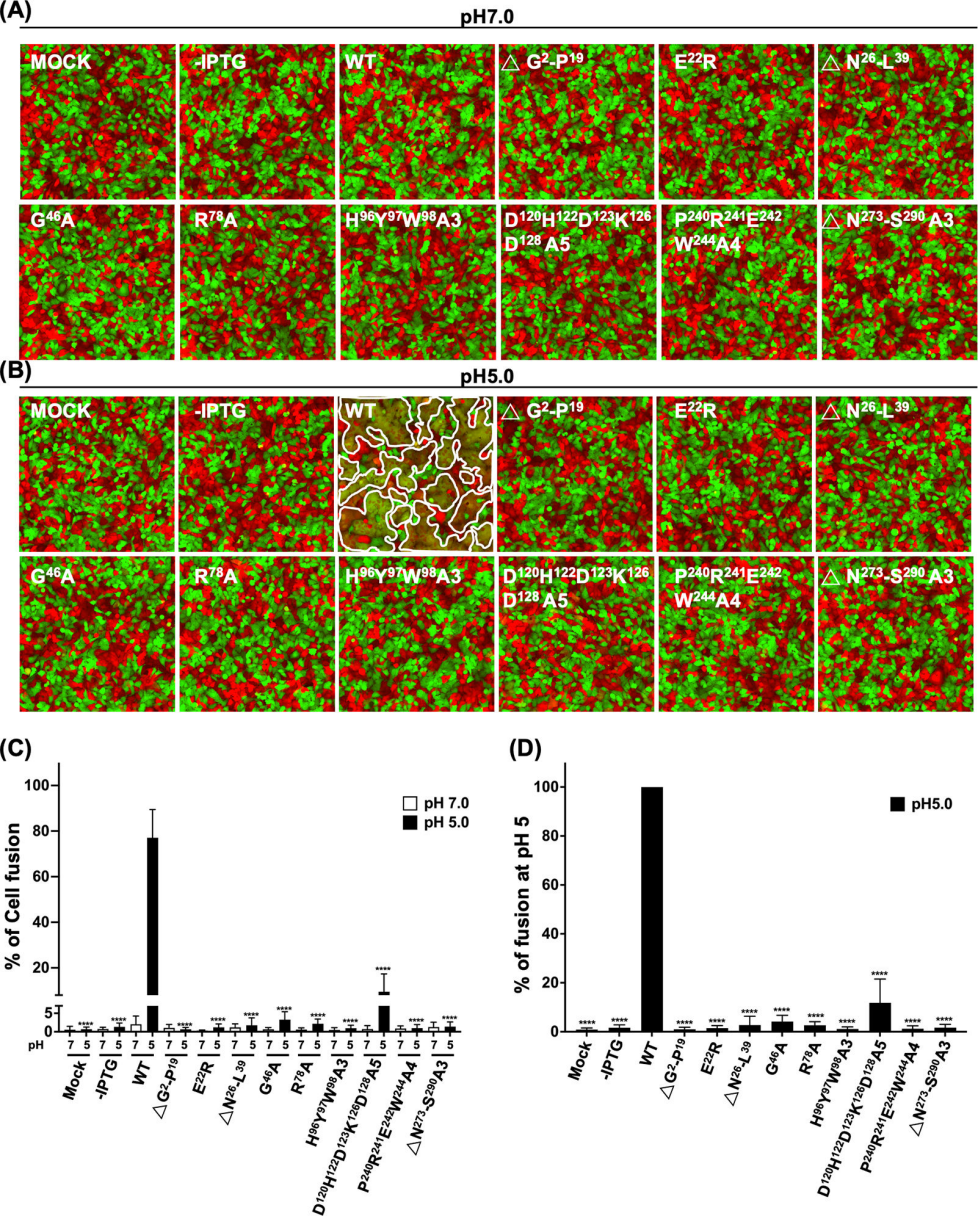
Cell-cell fusion assays of G9 mutants reveal impaired fusion activity at low pH. (A) HeLa cells expressing either GFP or RFP were mixed at a 1:1 ratio and incubated with lysates containing WT or mutant G9 viruses in neutral pH 7 buffer. Fluorescence images were captured at 3 h post-incubation. (B) Parallel fusion assays were performed as in (A), except in acidic pH 5 buffer. Areas outlined by white lines indicate fused cells. (C) Quantification of virus-induced cell-cell fusion at pH 5 and 7. Images from three independent experiments, as described in (A) and (B), were analyzed using Fiji software. Percent fusion was calculated as: (surface area of GFP^+^RFP^+^ double-fluorescent cells/surface area of single-fluorescent cells) × 100%. White bars indicate pH 7; black bars indicate pH 5. (D) Relative fusion efficiency of each mutant compared to WT G9 at pH 5. WT G9-triggered fusion at pH 5 (shown in C) was defined as 100%, and fusion efficiencies for each mutant were normalized accordingly. Data represent means ± standard deviations from three independent experiments. Statistical comparisons of fusion efficiency were performed between WT and each mutant using Student’s *t*-test: *P < 0.05; **P < 0.01; ***P < 0.001; and ****P < 0.0001.

Previous studies using fluorescence dequenching analyses showed that G9-deficient vaccinia MV particles failed to initiate hemifusion with host membranes (31). To explore whether the critical residues we identified above are also required for fusion activity, we performed MV-triggered cell-cell fusion assays using virus-containing lysates from the trans-complementation experiments as previously described (35, 36, 39). It is known that repression of vaccinia EFC gene expression did not impair virion morphogenesis and MV particles of normal morphology were produced from the infected cells (15, 16, 18, 20–22, 24, 25, 36). Consistently, our G9 mutants did not impair MV morphogenesis (Fig. S1), validating the use of these inf/tf lysates for MV-triggered cell fusion assays. HeLa cells expressing GFP or RFP were mixed at a 1:1 ratio and incubated with WT or mutant G9 virus lysates, treated with neutral pH 7 or acidic pH 5 buffer, and MV-triggered cell-cell fusion was monitored. As expected, no cell-cell fusion was triggered by WT nor mutant G9 viruses at neutral pH (Fig. 2A). As expected, WT G9 virus triggered robust cell-cell fusion under acidic conditions, while none of the nine G9 mutants triggered detectable fusion (Fig. 2B). Quantification of MV-triggered cell fusion at the neutral and acid pH was shown (Fig. 2C). The relative fusion rate to WT G9 at low pH revealed that all nine G9 mutants lacked membrane fusion activity (Fig. 2D), indicating these residues are essential for EFC-mediated membrane fusion.

### Defective Group 1 G9 mutants disrupted G9-A16 subcomplex and EFC assembly

**Fig 3:**
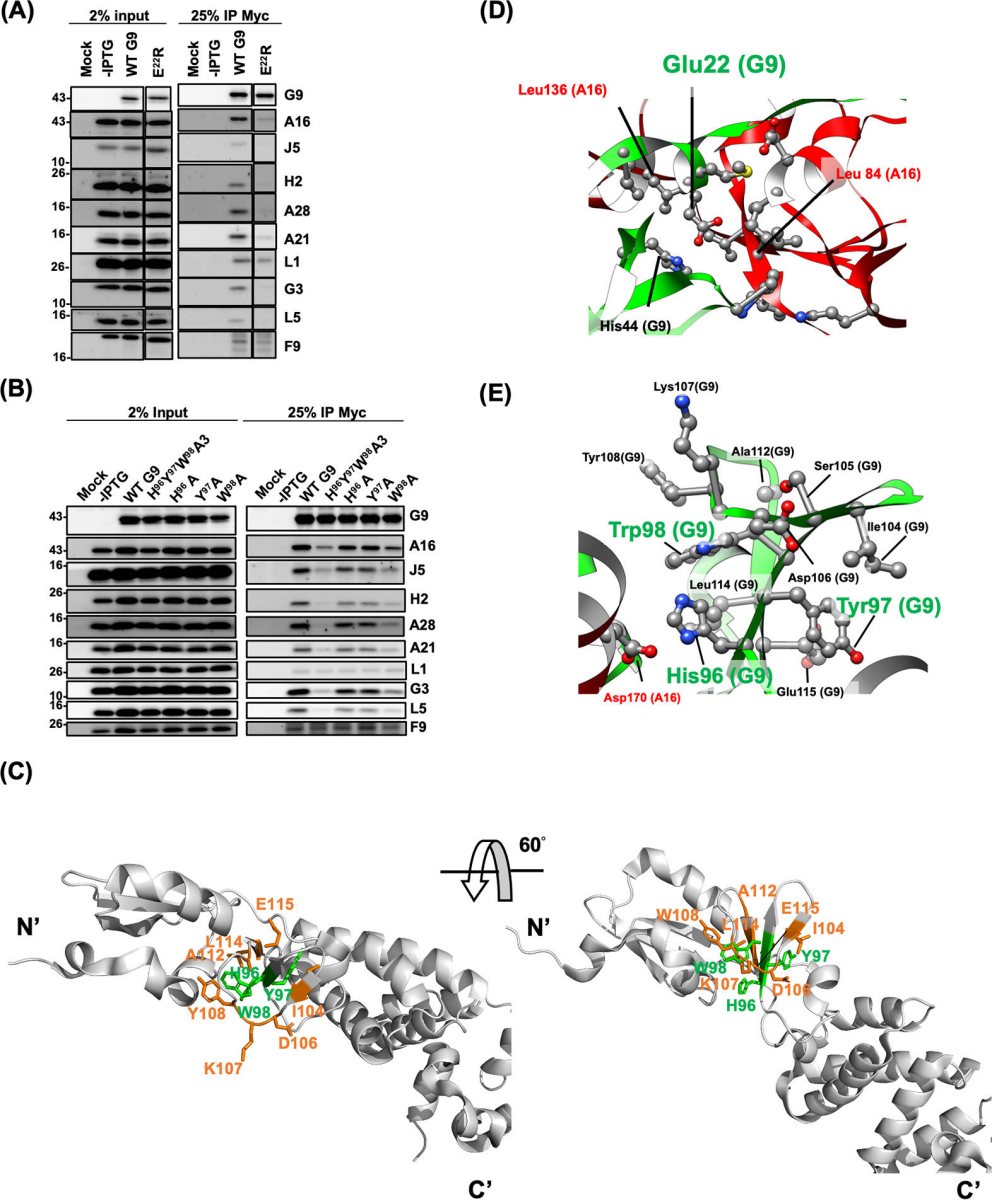
Group 1: G9 mutants deficient in binding A16 and other EFC components. (A and B) Co-immunoprecipitation (co-IP) of lysates from infected/transfected cells expressing G9 mutants. Two mutants, E^22^R (A) and H^96^Y^97^W^98^A3 (B), failed to co-precipitate A16 or other EFC proteins. (C) Structural context of the conserved H96Y97W98 motif (green), which forms hydrogen bonds, van der Waals, and hydrophobic contacts with nearby residues I104, D106, K107, Y108, A112, and L114 and E115 (orange). This triple β-strand motif likely serves as a linker bridging the N-terminal helical domain (residues 1–81) and the C-terminal region (residues 117–340) of G9. (D and E) Structural mapping of key residues based on the G9-A16 co-crystal structure (38). Critical residues on G9 (green) and their interacting partners on A16 (red) are highlighted in both cartoon and ball-and-stick representations, illustrating their spatial arrangement.

**Fig 4:**
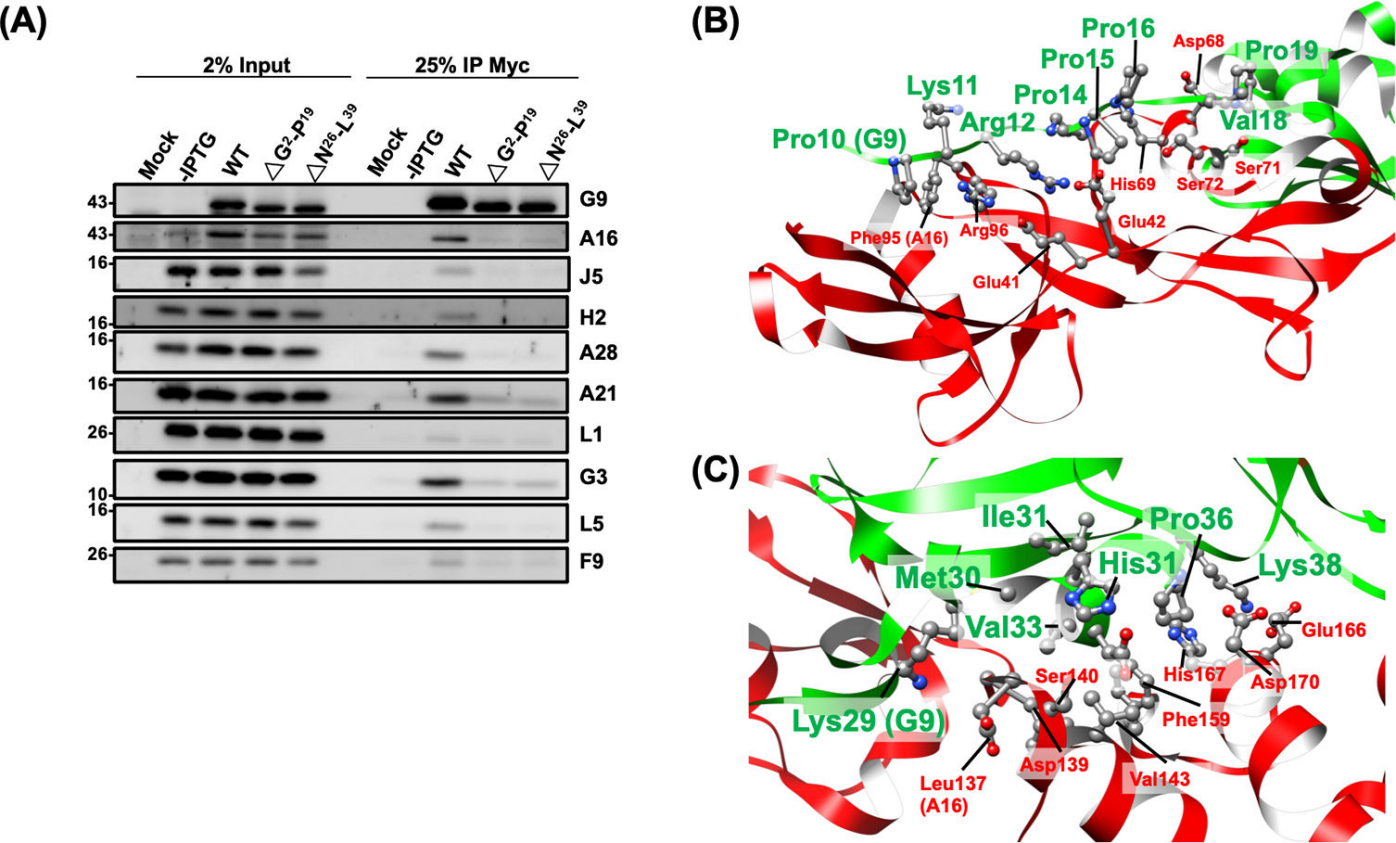
Group 1: N-terminal deletion mutants of G9 were unable to bind A16 or other EFC components (A) Co-IP of lysates from infected/transfected cells expressing G9 mutants ΔG^2^-P^19^ and ΔN^26^-L^39^. (B and C) Structural mapping of key residues based on the G9-A16 co-crystal structure (38). Critical residues on G9 (green) and their interacting partners on A16 (red) are highlighted. Cartoon and ball-stick formats depict the spatial relationship of these residues.

To investigate the molecular basis of fusion and infectivity defects, we performed co-immunoprecipitation (co-IP) experiments to assess whether G9 mutants interact with A16 protein and other EFC components. The results in Fig. 3 and 4 showed that the first four G9 mutants (named as the Group 1 mutants), G9^E22R^ (Fig. 3A and Fig. S2A), G9^H96Y97W98A3^ (Fig. 3B), G9^ΔG2-P19^, and G9^ΔN26-L39^ (Fig. 4A) failed to co-precipitate with A16 as well as other EFC components when the WT G9 protein co-precipitated all EFC components well (Fig. 3A and B and Fig. 4A).

We then looked at the locations of these important residues in the crystal structure of G9-A16 subcomplex (38) (Fig. 3C through E) and noticed that E22 is highly conserved in all the G9 orthologues in *Poxviridae* (Fig. 1A). Based on the Residue Interaction Network Generator (RING) analyses, E22 forms hydrogen and van der Waals (VDW) interactions with L84 and L136 of A16 protein, respectively (Fig. 3D). In addition, E22 is proximal (~3.4 Å) to H44 of G9 protein, suggesting a pH-dependent electrostatic interaction that may stabilize G9 conformation at low pH. The G9^E22R^ mutation likely introduces charge repulsion with H44, disrupting the pH-dependent interaction, implying that the E22-H44 interaction at low pH also contributes to G9 protein function.

The conserved G9 residues H96, Y97, and W98 form a β-strand, in which H96 interacts with D170 of the A16 protein through ionic and VDW interactions (Fig. 3E). Notably, Y97 and W98 engage in intra-molecular interactions with multiple residues of the G9 protein itself (I104, D106, K107, Y108, A112, L114, and E115) via hydrogen bonding, VDW forces, and hydrophobic interactions (Fig. 3C and E). These interactions collectively shape the region into a triple β-stranded structure flanked by a loop (Fig. 3C). Structurally, this region serves as a linker between the N-terminal helical domain (residues 1–81) and the C-terminal region (residues 117–340) of G9 (Fig. 3C). Consequently, the triple mutant G9^H96Y97W98A3^, which lacks the side chains necessary for these stabilizing interactions, likely disrupts the integrity of this linker domain, leading to structural alterations and functional loss of G9 protein.

In Fig. 4B, the deletion mutant G9^ΔG2-P19^ removed an N-terminal loop on G9 which is essential for contacting with the N-terminal region of A16 protein (E41, E42, D68, H69, S71, S72, F95, and R96). Consistent with this structure feature, the G9^ΔG2-P19^ mutant failed to interact with A16 and other EFC components (Fig. 4B). Finally, the N26-L39 region of G9 protein was previously shown to be important for superinfection interference by A56 and K2 proteins (41). Here, the co-IP results and the structure interpretation of the G9^ΔN26-L39^ mutant (Fig. 4A and C) showed that this region is important for interactions with multiple residues (L137, D139, S140, V143, F159, E166, H167, and D170) of A16 protein (Fig. 4C). Taken together, all the residues defined in the above-mentioned Group 1 mutants are important for G9 and A16 subcomplex interaction as well as the EFC formation.

### Defective Group 2 G9 mutants bound to A16 but failed to form EFC

**Fig 5:**
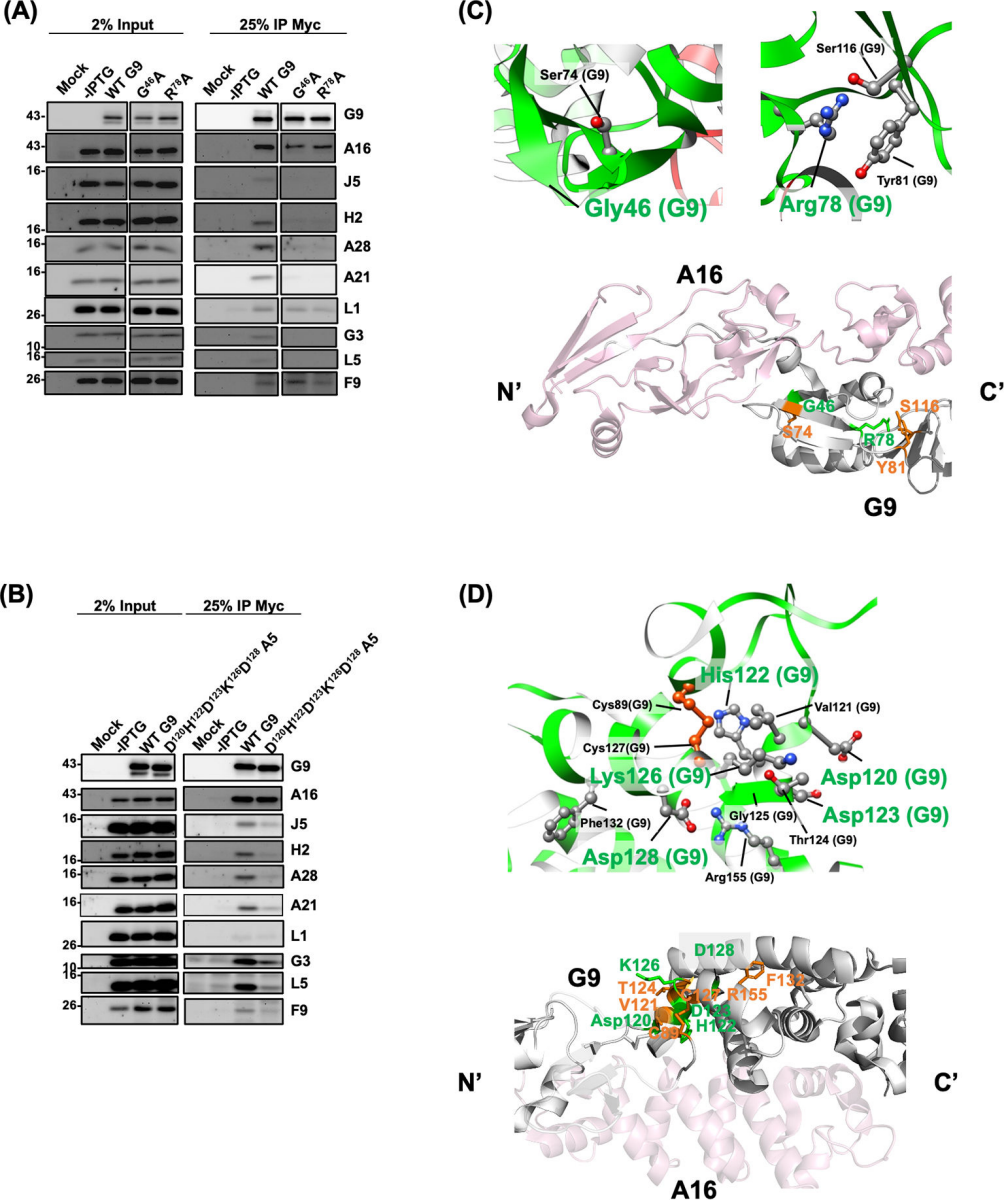
Group 2: G9 Mutants bound to A16 but failed to assemble a full EFC. (A and B) Co-IP assays showing G9 mutants (G^46^A, R^78^A [in A] and D^120^H^122^D^123^K^126^D^128^A5 [in B]) retained binding to A16 but failed to associate with other EFC components. (C) Structural insights into these residues, which are distal to the G9-A16 interface (38). (Top panel) The critical residues (green) on G9 are involved in intra-G9 interactions important for EFC assembly. The structures were presented in a flat-ribbon cartoon model, and the interaction side-chains were shown in ball and stick format. (Bottom panel) G46 and R78 (green) form internal contacts with S74, Y81, and S116 (orange) within G9 (gray ribbon), but do not directly contact A16 (pink ribbon) in the G9-A16 complex. (D) Structural insights into these residues, which are distal to the G9-A16 interface (38). (Top panel) The critical residues (green) on G9 are shown as described in (C). (Bottom panel) The structures were presented as described in (C). The surface cluster D120, H122, D123, K126, and D128 (green) interacts with adjacent residues V121, G125, C127, F132, and R155 (orange) but remains spatially distant from A16 (pink).

Group 2 mutants, G9^G46A^, G9^R78A^ (Fig. 5A and Fig. S2B), and G9^D120H122D123K126D128A5^ (Fig. 5B) retained A16 binding but failed to co-immunoprecipitate other EFC components. Structurally, the mutated residues in this group are located within internal regions of the WT G9 protein and appear to contribute to intra-molecular stabilization. For instance, G46 forms a hydrogen bond with S74, and R78 engages in VDW interactions with S116 and Y81 (Fig. 5C, upper panel). Likewise, the clustered mutations in G9^D120H122D123K126D128A5^ disrupt multiple internal contacts, including hydrogen bonding (involving D120, H122, D123, G125, D128, and F132), VDW interaction (V121 and C127), and ionic interaction (D128 with R155) (Fig. 5D, upper panel). Spatially, both G46 and R78 are located near the N-terminal region of G9 and are not in close contact with A16 (Fig. 5C, bottom panel), while the charged cluster (D120H122D123K126D128) lies on the protein surface but remains distal to A16 (Fig. 5D, bottom panel). These observations suggest that Group 2 mutations may impair EFC assembly by destabilizing G9’s tertiary structure, while preserving the G9-A16 subcomplex.

### Defective Group 3 G9 mutants, which harbor mutations in the conserved P(R/Y)XCW motif and/or the adjacent loop, retained A16 binding but failed to assemble the EFC

**Fig 6:**
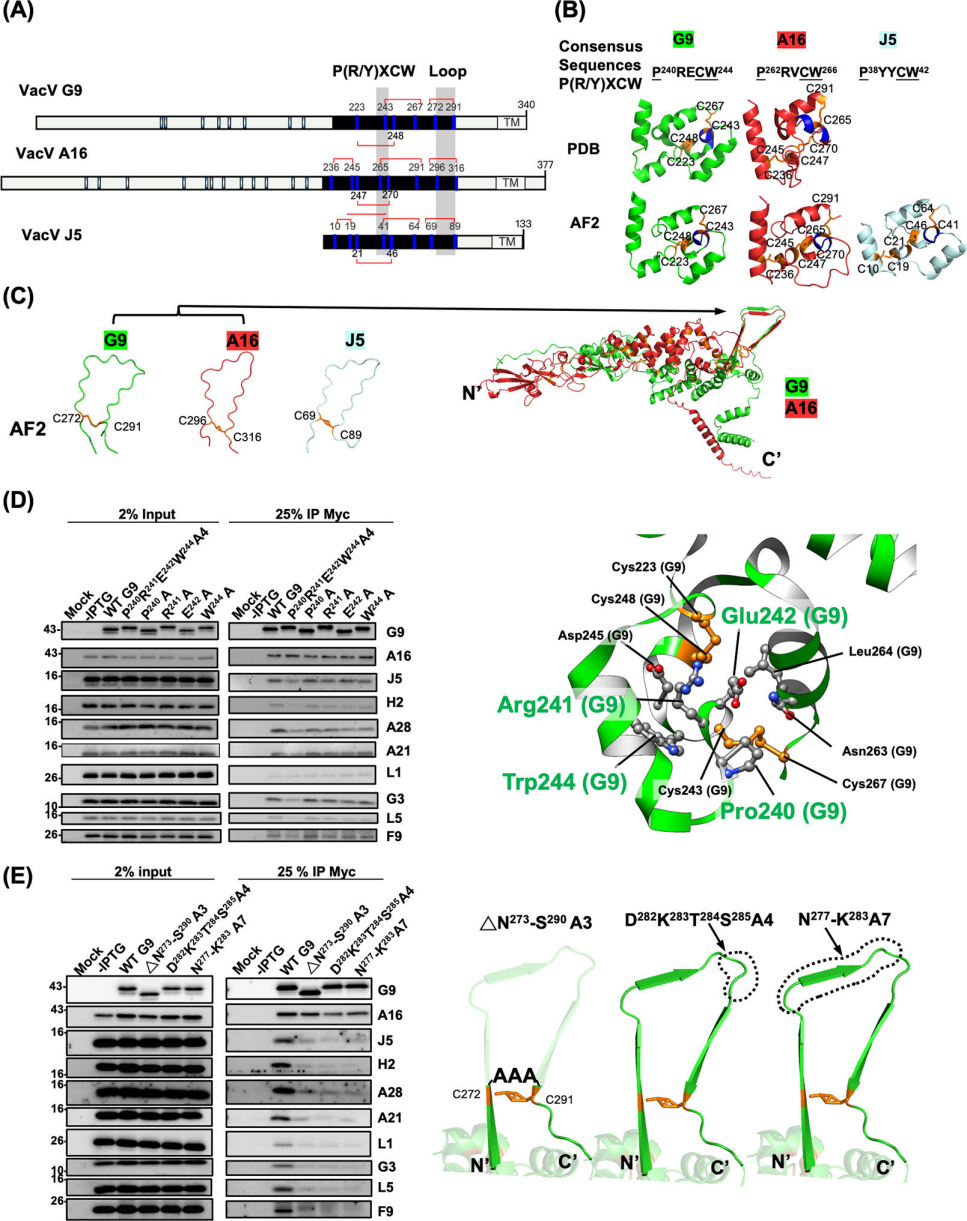
Group 3: conserved C-terminal G9 mutants affected EFC assembly. (A) Schematic representation of vaccinia G9, A16, and J5 protein. Cysteine residues are marked, and those within the G9/A16/J5 conserved region (40) (black) were numbered. The disulfide bonds (red lines) in the conserved P(R/Y)XCW and loop regions (shaded in gray) are shown. The scheme was generated with illustrator of biological sequences (IBS) (42). (B) The conserved P(R/Y)XCW motif (the helix marked in blue) in the G9 and A16 crystal structures (PDB) or in the J5 predicted structure from AlphaFold2 modeling (AF2) is shown. The conserved disulfide bonds are shown in orange. (C) The loop regions formed by disulfide bonds (in orange) in G9, A16, and J5 proteins are individually modeled by AF2 as distorted structure, whereas they are modeled as three β-strands in the full-length G9-A16 subcomplex. (D) Co-IP analyses of the tetra-mutant G9 ^P240R241E242W244A4^ and individual mutants G9^P240A^, G9^R241A^, G9^E242A^, and G9^W244A^ (the left panel). Structural insights of these target residues (in green) in G9 protein (the right panel). (E) Co-IP analyses of three loop mutants, G9^ΔN273-S290A3^, G9^D282K283T284S285A4^, and G9^N277-K283A7^ (left panel). The mutation designs in the G9 loop region are illustrated (right panel).

Next, as shown in Fig. 6, the last group of G9 mutants, named as the Group 3 mutants, contained G9^P240R241E242W244A4^ and G9^ΔN273-S290A3^ mutants. Both G9 mutants targeted multiple residues within a region that shared conserved disulfide bonding patterns among vaccinia G9, A16, and J5 proteins (red lines in Fig. 6A). Two interesting motifs, the P(R/Y)XCW that is surrounded by four to five conserved α-helical arrangements (Fig. 6B) and three homologous loops linked by disulfide bonds, are in this region (area shaded in gray in Fig. 6A). The latter motif was not in the G9-A16 complex crystal structure (38), and the comparable region in A16, G9, and J5 was predicted with AlphaFold2 as a closed loop via disulfide bonding (Fig. 6C, left panel). Furthermore, the G9-A16 complex structure covers only truncated forms of the proteins (38). To better understand the interactions within the full-length G9-A16 subcomplex, we employed AlphaFold2 to predict the complete structures in subcomplex form, which predicted that the loops form parallel β-strands at the G9-A16 interface (Fig. 6C, right panel). The average confidence scores for G9, A16, and J5 were high (pLDDT > 70; Fig. S3), although the loop regions showed lower confidence, as highlighted by the gray-shaded areas (Fig. S3). To assess the accuracy of the AlphaFold2 multimer prediction, we examined the pTM score. The G9-A16 subcomplex yielded a pTM score of 0.752, indicating high confidence in the overall fold predicted by AlphaFold2. We thus constructed two G9 mutants, including a tetra-mutant G9^P240R241E242W244A4^ that eliminated the P(R/Y)XCW and the G9^ΔN273-S290A3^ mutant that deleted the loop.

The co-IP results of the tetra mutant G9^P240R241E242W244A4^ retained the A16 binding but failed to co-IP other EFC components (left panel of Fig. 6D). Individual point mutations in this motif (G9^P240A^, G9^R241A^, G9^E242A^, and G9^W244A^) showed milder effects, suggesting that multiple side chains cooperate structurally (left panel of Fig. 6D). Based on the G9-A16 complex structure, P240, R241, E242, and W244 are not located at the interface of the G9-A16 subcomplex and, instead, they formed a compact helix via multiple self-interactions such as H-bonding between R241-W244 and P240-C243; VDW interactions between E242, N263, and L264. The PRECW-containing helix is also surrounded by three adjacent α-helices via interactions with N263 and L264, in addition to the described-above self-interacting residues. Taken together, these intra-molecular interactions work cooperatively to facilitate a stable PRECW motif in G9 protein (right panel of Fig. 6D).

Similarly, the G9^ΔN273–S290A3^ mutant, in which the loop spanning residues N273 to S290 was deleted and replaced with three alanine, retained A16 binding but lost interaction with other EFC components (Fig. 6E, left panel). To determine which portion of this loop is functionally important, we generated two substitution mutants, G9 ^D282K283T284S285A4^ and G9 ^N277-K283A7^ (Fig. 6E). Both mutants exhibited the same phenotype as the G9^ΔN273–S290A3^ mutant, losing virus infectivity but maintaining A16 binding while failing to recruit other EFC proteins. These findings demonstrate that this conserved loop is essential for complete EFC assembly and highlight the critical role of Group 3 residues in stabilizing the fusion-competent architecture of the G9 protein.

## DISCUSSION

**Fig 7:**
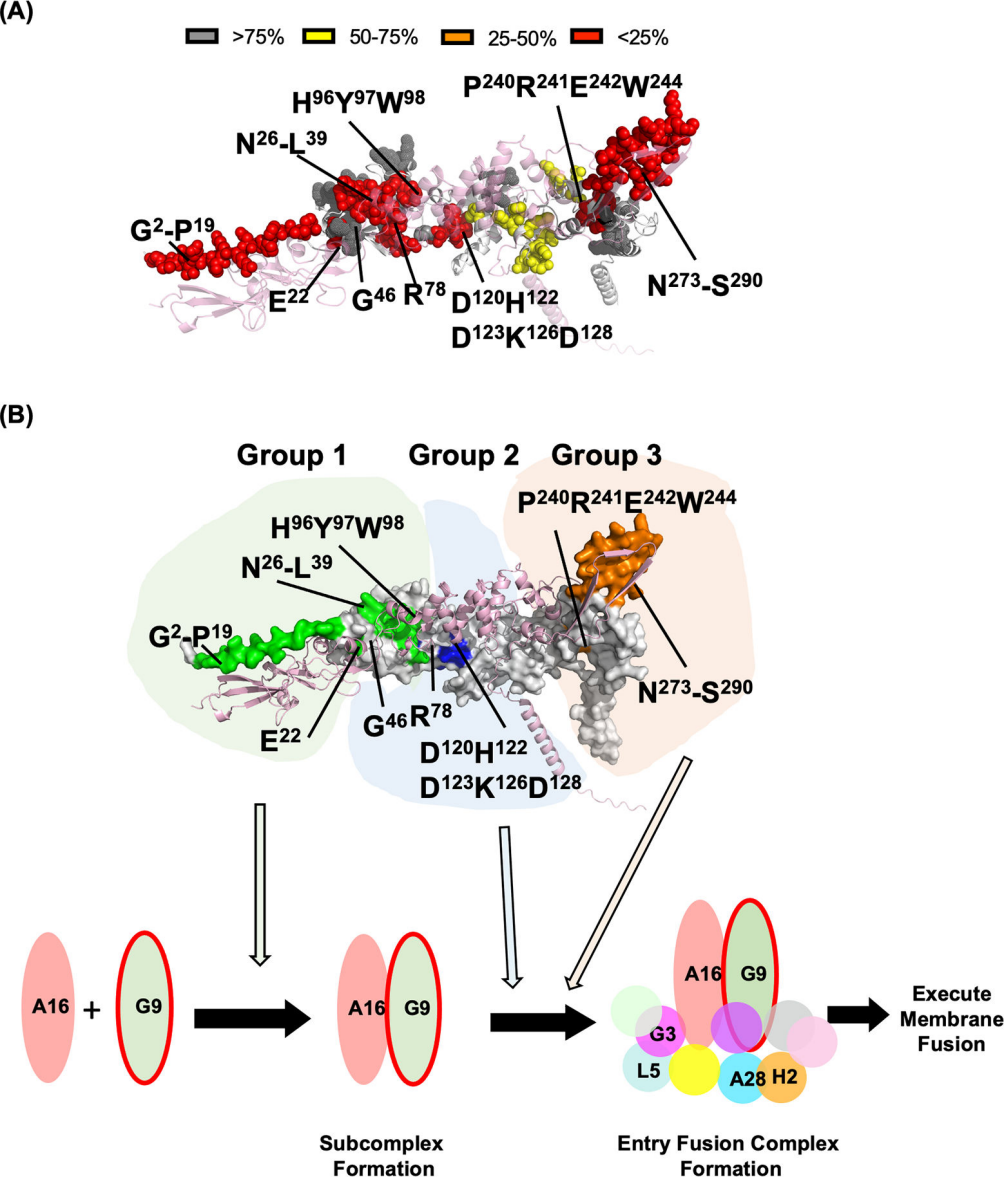
Summary of functionally important regions in vaccinia G9 protein. (A) The full-length G9-A16 AF2-predicted model colored with G9 residues that, when mutated, exhibited a significant loss of MV infectivity: mutant titer <25% of WT (in red), ~25–50% of WT (in orange), ~50–75% of WT (in yellow), and >75% of WT (in gray). VacV A16 protein is shown in pink. (B) The overall working model illustrating G9’s Group 1 residues involved in G9-A16 subcomplex formation, while Groups 2 and 3 residues facilitate the subsequent EFC formation.

In this study, we performed a functional analysis of the vaccinia virus G9 protein and identified residues critical for virus infectivity (Fig. 1B), MV-mediated membrane fusion (Fig. 2), and EFC formation (Fig. 3 through 6). We summarized our results in Fig. 7A, where residues essential for G9 function are color-coded by severity (red, orange, and yellow). Based on co-immunoprecipitation data, we classified these residues into three functional groups (Fig. 7B), which reflect their contributions to G9-A16 subcomplex formation and broader EFC assembly.

We examined 47 mutations covering approximately 34% of G9 protein sequences. The nine important G9 mutants are classified into three groups. The Group 1 mutants, G9^E22R^, G9^H96Y97W98A3^, G9^ΔG2-P19^, and G9^ΔN26-L39^ directly interrupted G9 and A16 protein-protein interaction and as well as EFC formation (green area, Fig. 7B). The Group 2 mutant G9 proteins, including G9^G46A^, G9^R78A^, and the G9^D120H122D123K126D128A5^, bound to A16 protein but not to other EFC components (blue area, Fig. 7B). Finally, Group 3 G9^P240R241E242W244A4^ and G9 ^△N273-S290A3^ mutants are focused on two motifs that are conserved among G9, A16, and J5. Group 3 mutations, similar to Group 2, bound to A16 protein but not to other EFC components (orange, Fig. 7B). Both Group 2 and Group 3 mutations are far from the G9 and A16 interaction interphase, explaining why these mutations did not affect G9-A16 subcomplex formation. The mechanisms by which Group 2 and Group 3 mutations disrupt EFC assembly were unclear in the absence of a high-resolution EFC structure. However, while this manuscript was under review, our lab recently determined the cryo-EM structure of the EFC which indicated that the residues targeted in the Group 2 mutants are not involved in G9 interactions with other EFC components. Instead, these residues are buried within G9, contributing to domain folding and protein stability. Mutations at these positions may induce allosteric changes that destabilize the overall EFC assembly. Furthermore, the EFC structure revealed that the two conserved motifs in Group 3, the PRECW motif and the adjacent loop, are positioned at interfaces critical for heterotrimer formation among G9, A16, and J5. These insights suggest that Group 2 and Group 3 residues likely contribute to interactions between G9 and other EFC components beyond A16, in contrast to Group 1 residues at the N-terminus of G9, which primarily stabilize the G9-A16 subcomplex. At this point, our data implied that G9 and A16 subcomplex formation may be a prerequisite for the viral EFC assembly (bottom panel of Fig. 7B).

**Fig 8:**
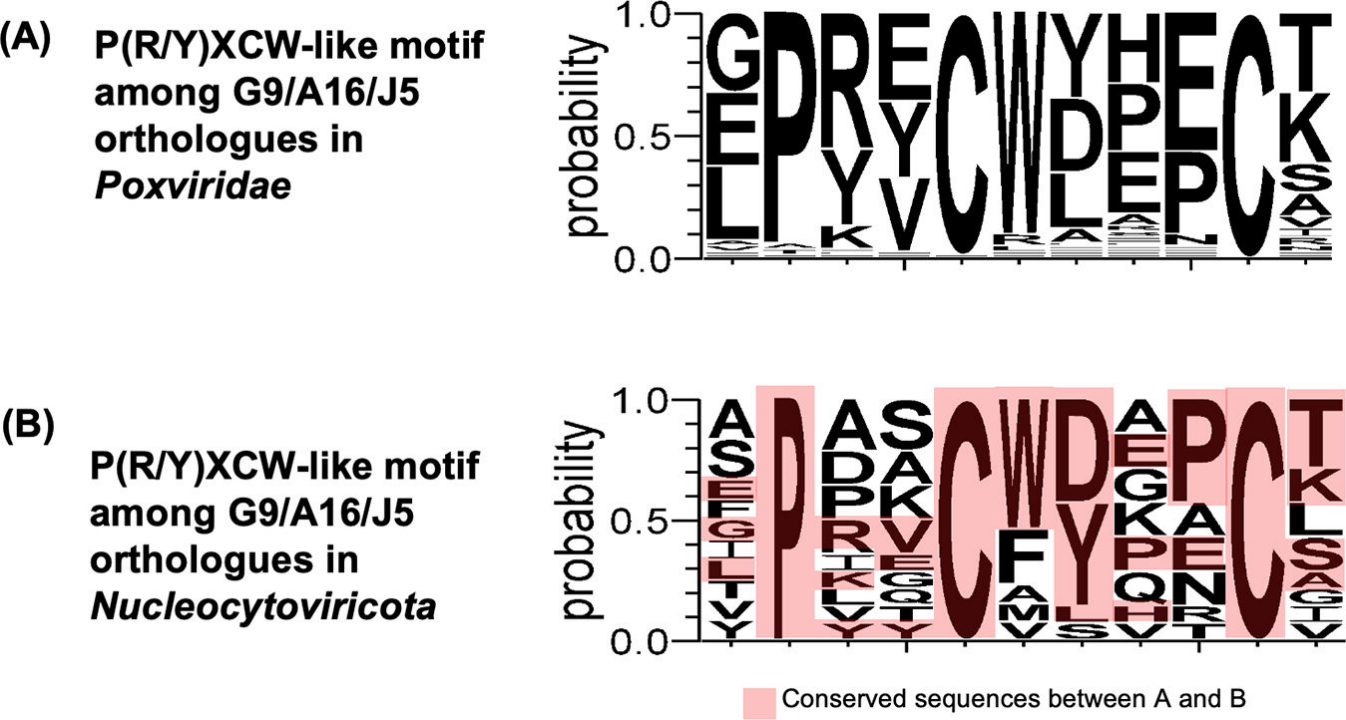
The P(R/Y)XCW motif is conserved across *Poxviridae* and *Nucleocytoviricota*. (A) Sequence logo generated from 28 *Poxviridae* members (described in reference 36) shows high conservation of the P(R/Y)XCW motif. (B) Alignment of 11 G9 homologous genes from six *Nucleocytoviricota* members (Klosneuvirus, Frog virus 3, Tupanvirus deep ocean, Medusavirus, Pacmanvirus A23, and Invertebrate iridescent virus 6) also reveals a conserved motif. The highly conserved sequence was labeled in pink.

Interestingly, in this study, we identified a functionally important motif, P(R/Y)XCW, within a conserved disulfide bonding region among vaccinia G9, A16, and J5 proteins (Fig. 6A and B). Sequence alignment of orthologs of G9, A16, and J5 in 28 Poxviridae members (e.g., VACV, CMLV, CPXV, ECTV, and MPXV) together revealed a highly conserved consensus sequence in this region (Fig. 8A). We previously showed that vaccinia G9 homologs are present in members of *Nucleocytoviricota* (40). We expanded the homology analysis and found that these G9 homologs in giant viruses also share this motif (Fig. 8B). These findings suggest that this conserved domain is a fundamental element of viral membrane fusion machinery and may have been evolutionarily retained among large DNA viruses.

The identification of critical residues in vaccinia G9 protein delineates distinct structural and functional roles within the EFC. Group 1 residues are indispensable for forming the G9-A16 subcomplex and serve as scaffolding elements for EFC nucleation. Group 2 and Group 3 residues, although not required for A16 interaction, are crucial for downstream complex assembly. Collectively, our results reveal that G9 contains multiple discrete domains that mediate sequential steps of EFC formation and membrane fusion. By identifying conserved and structurally vital residues, this study deepens our understanding of poxvirus EFC and identifies potential targets for antiviral strategies.

## Data Availability

The data sets generated during and/or analyzed during the current study are available from the corresponding author on reasonable request.

## SUPPLEMENTAL MATERIAL

Supplemental figures jvi.00723-25-s0001.pdf Figures S1 to S3.

ASM does not own the copyrights to Supplemental Material that may be linked to, or accessed through, an article. The authors have granted ASM a non-exclusive, world-wide license to publish the Supplemental Material files. Please contact the corresponding author directly for reuse.
