# Supplementary material for: Structural dissection of vaccinia G9 identifies residues essential for membrane fusion and complex assembly
Source: J Virol. 2025 Aug 18;99(9):e00723-25. doi: 10.1128/jvi.00723-25 (PMC12456145; doi:10.1128/jvi.00723-25)
Supplement: Supplemental figures — Figures S1 to S3. [file jvi.00723-25-s0001.pdf]

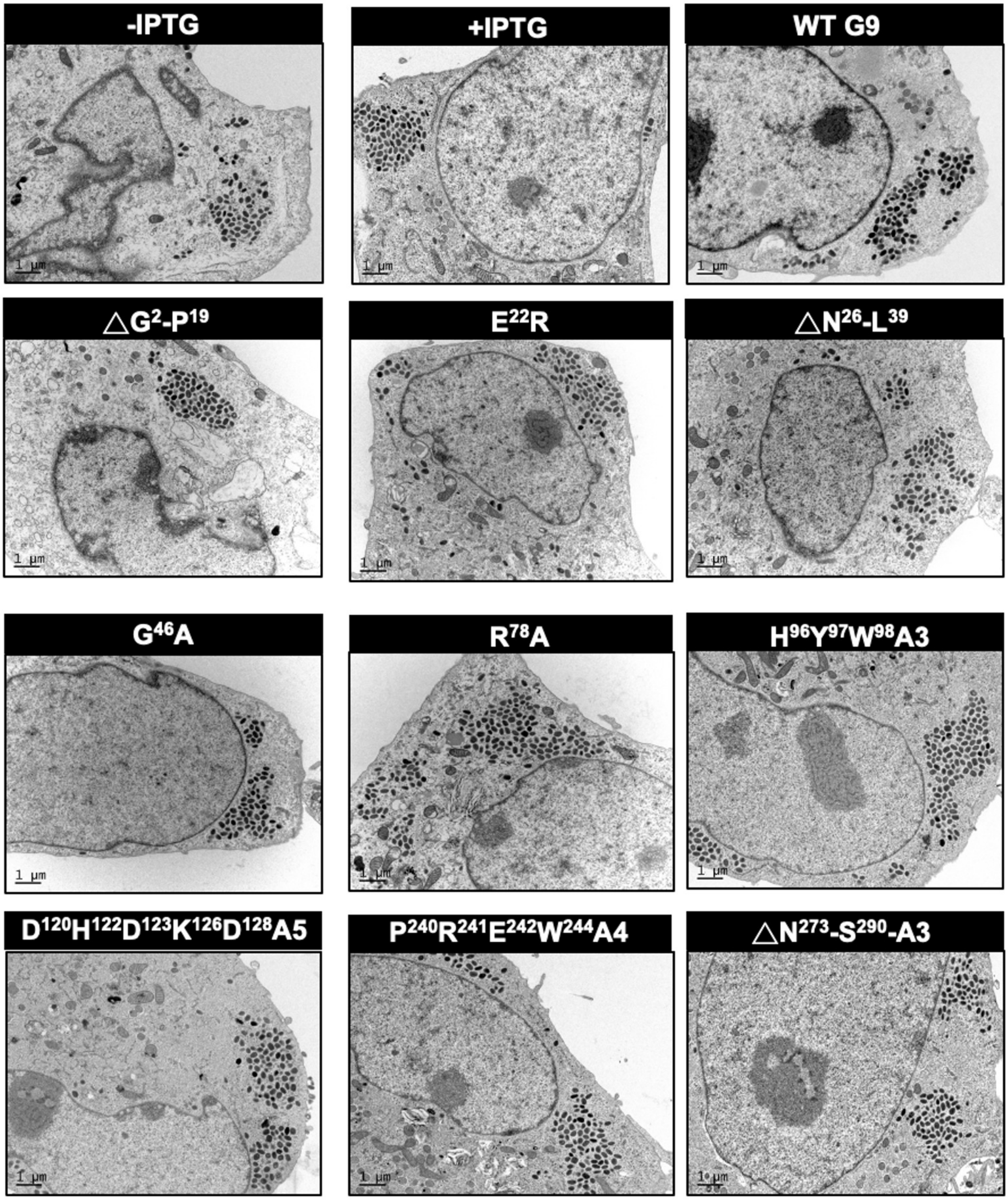

Fig. S1. Normal MV morphogenesis of G9 mutant viruses.
BSC40 cells were infected with vG9Li-HA (5 PFU/cell, 1 h at 37 °C), followed by transfection with either wild-type or mutant G9 plasmids. Infected/transfected cells were incubated in complete medium without IPTG for 24 h and processed for transmission electron microscopy as described in *Materials and Methods*. Scale bar, 1 μm.

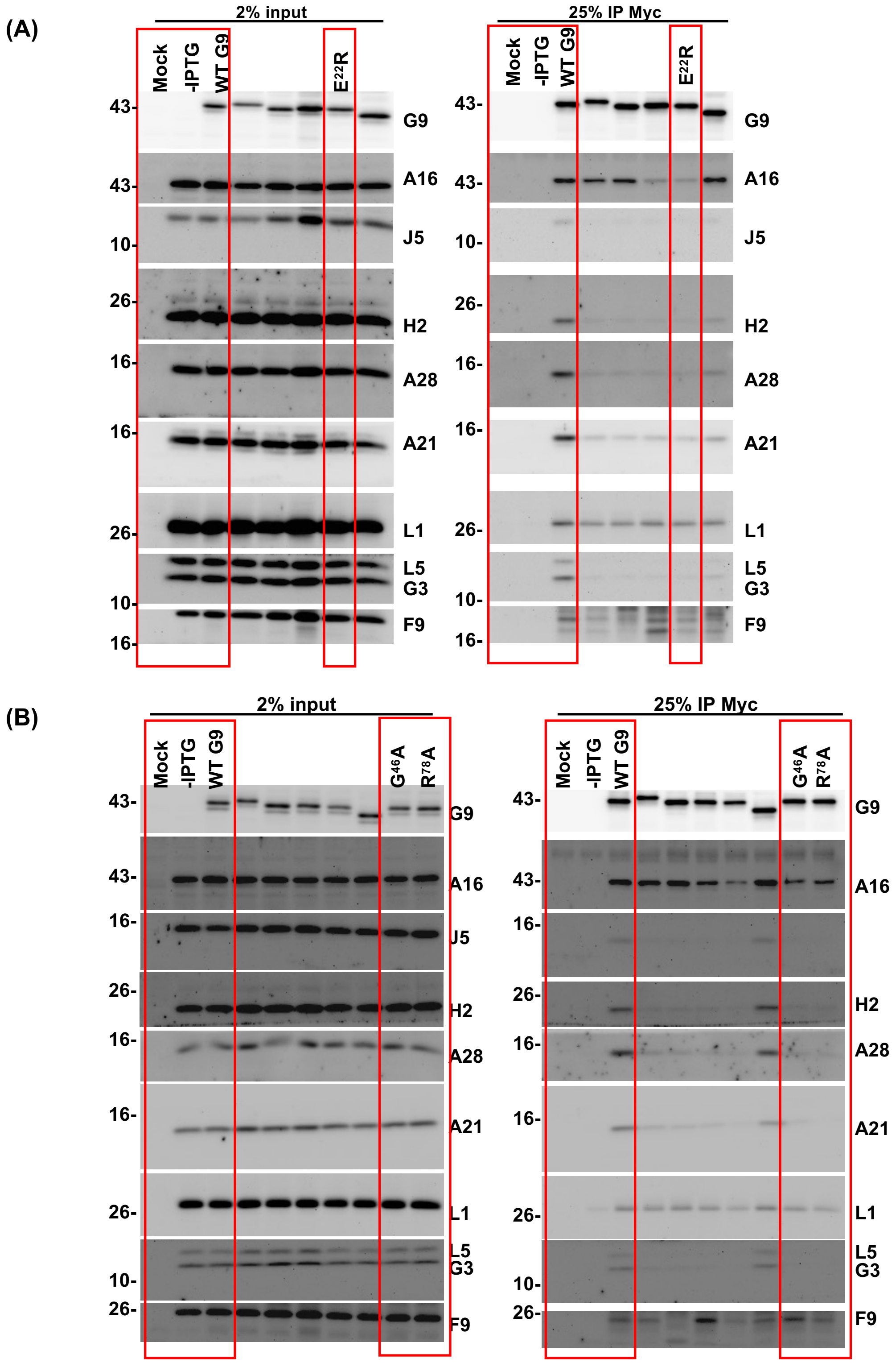

Fig. S2. Uncropped immunoblots referred to Figure 3A and Figure 5A.
The red boxes mark the lanes of immunoblots shown in Figure 3A (A) and Figure 5A (B).

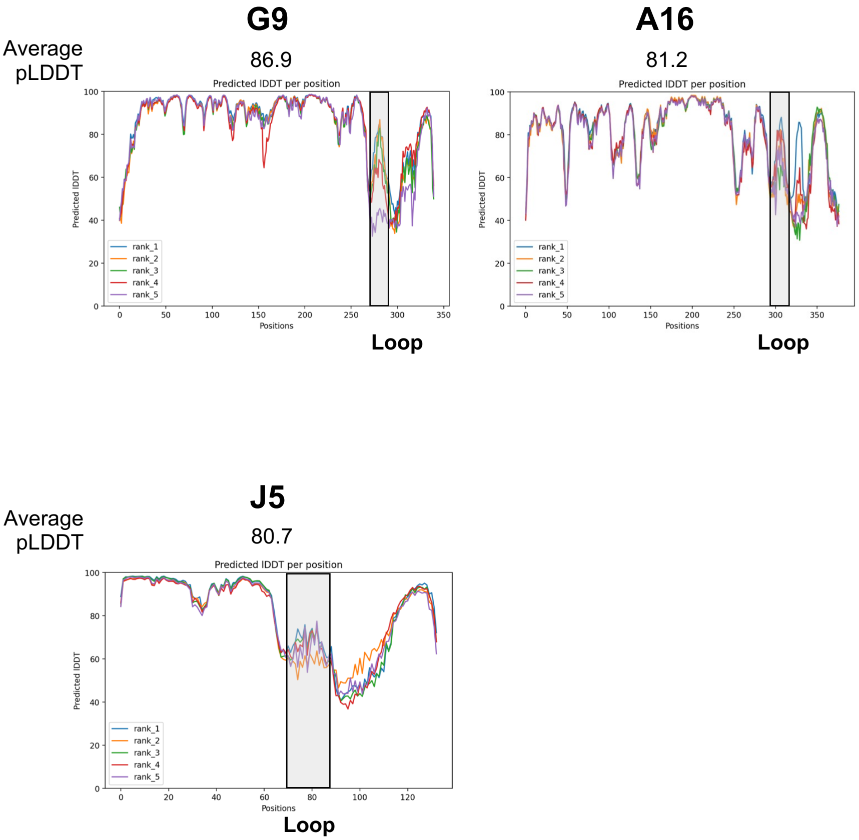

Fig. S3. The pLDDT score of the predicted A16, G9 and J5 models.
The predicted AF2 model with an average pLDDT score >70 was selected for each protein. Final confidence scores for G9, A16 and J5 are 86.9, 81.2 and 80.7, respectively. The gray square labeled the conserved loop region in G9, A16, and J5.
